# A Meta-Analysis Based Method for Prioritizing Candidate Genes Involved in a Pre-specific Function

**DOI:** 10.3389/fpls.2016.01914

**Published:** 2016-12-15

**Authors:** Jingjing Zhai, Yunjia Tang, Hao Yuan, Longteng Wang, Haoli Shang, Chuang Ma

**Affiliations:** State Kay Laboratory of Crop Stress Biology for Arid Areas, College of Life Sciences, Northwest A&F UniversityYangling, China

**Keywords:** biological network, data fusion, flowering time, gene prioritization, machine learning, meta-analysis, rank aggregation, systems biology

## Abstract

The identification of genes associated with a given biological function in plants remains a challenge, although network-based gene prioritization algorithms have been developed for *Arabidopsis thaliana* and many non-model plant species. Nevertheless, these network-based gene prioritization algorithms have encountered several problems; one in particular is that of unsatisfactory prediction accuracy due to limited network coverage, varying link quality, and/or uncertain network connectivity. Thus, a model that integrates complementary biological data may be expected to increase the prediction accuracy of gene prioritization. Toward this goal, we developed a novel gene prioritization method named RafSee, to rank candidate genes using a random forest algorithm that integrates sequence, evolutionary, and epigenetic features of plants. Subsequently, we proposed an integrative approach named RAP (Rank Aggregation-based data fusion for gene Prioritization), in which an order statistics-based meta-analysis was used to aggregate the rank of the network-based gene prioritization method and RafSee, for accurately prioritizing candidate genes involved in a pre-specific biological function. Finally, we showcased the utility of RAP by prioritizing 380 flowering-time genes in *Arabidopsis*. The “leave-one-out” cross-validation experiment showed that RafSee could work as a complement to a current state-of-art network-based gene prioritization system (AraNet v2). Moreover, RAP ranked 53.68% (204/380) flowering-time genes higher than AraNet v2, resulting in an 39.46% improvement in term of the first quartile rank. Further evaluations also showed that RAP was effective in prioritizing genes-related to different abiotic stresses. To enhance the usability of RAP for *Arabidopsis* and non-model plant species, an R package implementing the method is freely available at http://bioinfo.nwafu.edu.cn/software.

## Introduction

A major challenge in plant biology is to identify the most promising genes from large lists of candidate genes (e.g., all genes in the whole genome) to find those which play an important role in an agricultural trait or a complex biological process (Lee et al., 2010; Li et al., 2015; Sabaghian et al., 2015). However, an experimental validation of every candidate gene is very time-consuming and costly. A biologist would have to manually select the promising genes based on their potential function, a difficult task considering the paucity and disparity of functional annotation in plant species (Rhee and Mutwil, 2014). Computational methods are thus required to help biologists automatically prioritize candidate genes by integrating large amounts of functional genomic data that is now publicly available.

Gene prioritization was first developed to identify disease-associated human genes within a multigene locus identified by a positional genetic study (Perez-Iratxeta et al., 2002). This application was subsequently expanded to studies that generate candidate genes from the whole genome using genome-wide association analyses and “–omics” experiments (Moreau and Tranchevent, 2012). A number of computational approaches and bioinformatics tools have been developed to prioritize disease-related human genes with the use of various data sources such as scientific texts, protein-protein interactions, and functional annotations or pathways (Tranchevent et al., 2011; Moreau and Tranchevent, 2012). However, to the best of our knowledge, none of these approaches and tools designed for human studies can be directly applied to tackle the gene prioritization problem in plants.

Although functional genomic data are becoming available for many plant species, gene prioritization is still nascent in plant science. Until recently, only a few computational algorithms had been developed to address the challenge of gene prioritization for the model plant *Arabidopsis thaliana* (Lee et al., 2010, 2015b; Ma et al., 2014; Sabaghian et al., 2015). Network-based gene prioritization is a commonly used strategy because it is capable of characterizing the complex relationships among genes. In addition to gene co-expression networks (Ma et al., 2014), integrated functional association networks have been recently developed to prioritize genes in plants (Lee et al., 2010, 2015a,b; Warde-Farley et al., 2010; Sabaghian et al., 2015). One of the better known functional association networks is AraNet (http://www.functionalnet.org/aranet), which was originally built for prioritizing genes for *Arabidopsis thaliana* using a modified Bayesian system for integrating 24 distinct types of gene-gene associations derived from plant and non-plant species (Lee et al., 2010). The power of AraNet in gene prioritization has been demonstrated by the identification of regulators of drought sensitivity and lateral root development in *Arabidopsis* (Lee et al., 2010). Given the importance of network-based gene prioritization in the identification of plant gene function and in the genetic analysis of plant traits, the integration of functional associations into the design of network-based gene prioritization system (e.g., AraNet v2; http://www.inetbio.org/aranet), has been implemented for 28 non-model plant organisms, including some important crops like *Zea mays* (MaizeNet; http://www.inetbio.org/maizenet) and *Oryza sativa* (RiceNet v2; http://www.inetbio.org/ricenet; Lee et al., 2015a).

The rapid increase of functional association networks could accelerate the discovery of genes that are involved in a specific biological process or associated with plant traits of interest. However, the performance of network-based gene prioritization is still unsatisfied, due to limited network coverage, varying link quality, and/or uncertain network connectivity (Lee et al., 2010, 2011). Hence, novel gene prioritization algorithms that integrate sequence, evolutionary, and epigenetic features would complement and strengthen network-based gene prioritization algorithms; this is because some sequence-based features are capable of predicting protein functions (Lee et al., 2009; Libbrecht and Noble, 2015; Lloyd et al., 2015). To summarize, there is a recognized need for developing novel gene prioritization algorithms capable of integrating different type of features, and for investigating how these integrative algorithms may complement the conventional network-based gene prioritization algorithms (e.g., AraNet v2).

In this study, we first develop a novel gene prioritization method named RafSee: it applies a random forest algorithm to integrate features from protein sequences, evolutionary conservation, and epigenetic methylation marks. We then propose an integrative approach named RAP: it prioritizes the most promising genes by aggregating the prediction results from the network-based gene prioritization algorithm and RafSee using an order statistics-based meta-analysis strategy (Kolde et al., 2012). We go on to evaluate the prioritization ability of RafSee, of RAP, and of one state-of-the-art network-based gene prioritization system (AraNet v2), using 449 known flowering-time-related *Arabidopsis* genes manually compiled from different sources. We show that RafSee could be used as a robust complement to AraNet v2 for the prioritization of flowering-time genes in *Arabidopsis*. Moreover, we show that RAP performs better than either AraNet v2 or RafSee in most cases. The RAP method has been implemented as an R package available for public use.

## Materials and methods

### Workflow of RAP

**Figure 1:**
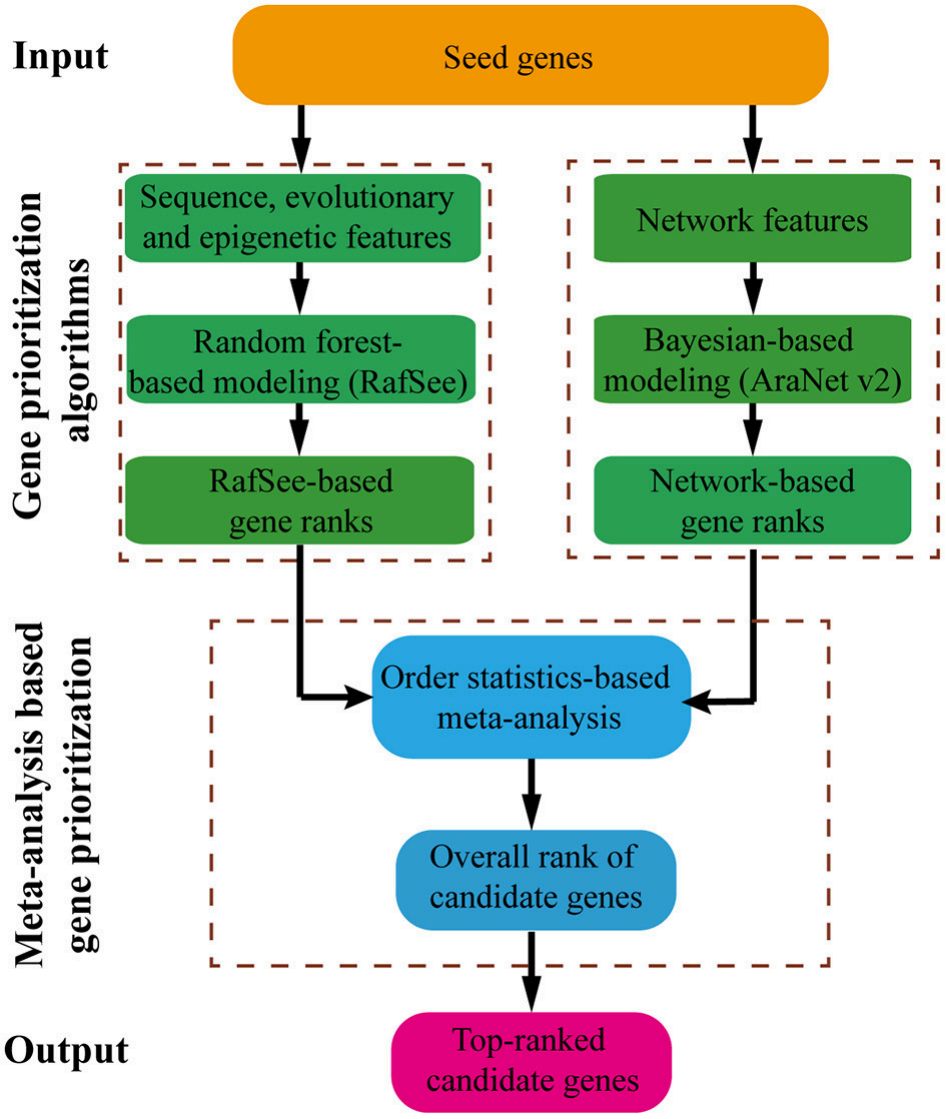
**Schematic of the RAP-based gene prioritization**.

The workflow of RAP is shown in Figure 1. Starting from a set of seed genes, RAP first builds an integrative random forest-based gene prioritization method (RafSee) using sequence, evolutionary, and epigenetic features. Then, using an order statistics-based meta-analysis approach, RAP aggregates prediction results from RafSee and one network-based gene prioritization system (AraNet v2) to deliver the top-ranked candidate genes for further experimental validation.

### Compilation of seed genes

To identify candidate flowering-time genes we used seed genes. The latter are a set of genes with a known function in flowering-time control which were collected from four different sources: (1) 293 flowering-time genes annotated in WikiPathways, which is an open, collaborative platform for the curation of pathways by researchers in the entire biology community (http://www.wikipathways.org/index.php/Pathway:WP2312; Kutmon et al., 2016); (2) 293 flowering-time genes collected by Zhu et al. (2011), according to the annotation related to flowering-related traits in The Arabidopsis Information Resource (TAIR) database (TAIR10; version 10; https://www.arabidopsis.org); (3) 406 flowering-time genes manually collected from literatures by Chen et al. (2012); (4) 174 flowering-time genes collected by the research group of Professor George Coupland at Max Planck Institute for Plant Breeding Research (http://www.mpipz.mpg.de/14637/Arabidopsis_flowering_genes). After eliminating 14 microRNA genes, we finally obtained a total of 449 protein-coding genes related to flowering time in *Arabidopsis* (Table S1).

### RafSee, an integrative random forest-based gene prioritization method

The main process for developing an integrative random forest-based gene prioritization method RafSee had four steps.

**Step 1: Sample labeling**. A total of 27 416 *Arabidopsis* genes annotated in the TAIR10 database were partitioned into three sample sets—positive, negative, and undocumented—of which the first two were required for training the random forest-based machine learning system. The positive sample set consisted of 449 known flowering-time genes (i.e., seed genes). The negative sample set consisted of 8503 protein-coding *Arabidopsis* genes, which had weak or no functional associations with the 449 flowering-time genes as annotated in the *Arabidopsis* protein interaction network from the STRING (Search Tool for the Retrieval of Interacting Genes/Proteins) database (version 10.0; http://string-db.org; 10 637 352 *Arabidopsis* protein interactions from 24 283 *Arabidopsis* proteins; downloaded on Feb. 25th, 2016). The STRING network was built based on the estimation of an association of confidence score for each protein pair using a Bayesian method that integrated various sources of interactions such as yeast two-hybrid experiments, text mining, co-expression, protein homology, etc. (Jensen et al., 2009). Two proteins were unlinked in the network when their association of confidence was weak (i.e., score <0.15). Note that some of the positive samples may be erroneously annotated as flowering-time genes, while some of the negative samples may in fact be true flowering-time genes not yet discovered. The remaining 18,464 protein-coding genes annotated for the *Arabidopsis* genome in the TAIR10 database were labeled as undocumented samples.

**Step 2: Feature encoding**. To be recognized by the random forest-based machine learning system, each protein sequence of 27,416 *Arabidopsis* genes was characterized by sequence, evolutionary, and epigenetic features, resulting in the generation of a 1012-dimensional feature vector with seven encoding schemes. The sequence-based features were generated with four encoding schemes, which are described in detail below.

- **Amino acid composition (AAC):** The AAC was a 420-dimensional numeric vector, which measured the occurrence frequency of 20 amino acids and 400 amino acid pairs in a protein sequence.
- **Pseudo amino acid composition (PAAC):** The PAAC incorporates both the composition of amino acids and their sequence-order information in a protein (Chou, 2001). There were 25 PAAC-related numeric features generated using the R package “protr” (version 1.1-1; https://cran.r-project.org/web/packages/protr; Zhang et al., 2013) with the parameters λ = 5, ω = 0.05. The first 20 features are associated with the occurrence frequency of the 20 amino acids, whereas the next five features (21–25) reflect the effect of sequence order (see Supplementary Data 1 for full details).
- **Amphiphilic pseudo amino acid composition (APAAC):** A total of 30 APAAC-related numeric features were generated using the R package “protr” with the parameters λ = 5, ω = 0.05. The first 20 features reflect the components of 20 amino acids, whereas the additional 10 features are a set of correlation factors that represent different hydrophobicity and hydrophilicity distribution patterns along a protein sequence (see Supplementary Data 1 for full details).
- **Physicochemical properties (PCPs):** For each amino acid, 533 PCPs were generated to describe various physicochemical properties using the R package “Interpol” (version 1.3.1; https://cran.r-project.org/web/packages/Interpol). The score matrix of PCPs (533 PCPs in rows, 20 amino acids in columns) is given in Table S2. As described in Jeong et al. (2009), for a given protein with a sequence length L, the normalized value for a specific physicochemical property *j* was calculated using the formula: $P(j)=\frac{1}{L}\sum_{i\;=\;1}^{L}\frac{p_{i}^{j}-p_{min}^{j}}{p_{max}^{j}-p_{min}^{j}}$, where $p_{i}^{j}$ is the score of property *j* for the residue at position *i*, $p_{max}^{j}$ and $p_{min}^{j}$ are the maximum and minimum values of the property *j*, respectively. The PCP-based encoding scheme generated a total of 533 numeric features for the corresponding physicochemical properties.

The evolutionary-based features included a sequence conservation (SC)-related feature and two whole genome duplication (WGD)-related features. The SC-related feature compares the sequence identity of *Arabidopsis* protein sequences to protein sequences from other 34 plant species—26 dicotyledonous, six monocotyledonous, and two other embryophyte species. For a given *Arabidopsis* gene, the BLASTP (basic local alignment search tool for proteins; http://blast.ncbi.nlm.nih.gov/Blast.cgi) similarity search was first performed to compare the protein sequence with those from 34 other plant species. Then, the 34 identities of the best BLASTP matches in the corresponding plant species were selected. Finally, a SC value was assigned as the median value of these 34 identities. For the two WGD-related features, two binary values were used to indicate either the presence (1) or absence (0) of a paralog produced in α and βγ WGD events. Genes with paralogs derived from α and/or βγ WGD events were identified by Bowers et al. (2003). These three evolutionary-based features have recently been encoded for the identification of essential genes in *Arabidopsis* (Lloyd et al., 2015).

The epigenetic feature is a binary value indicating whether the gene body is methylated or not. Body-methylated genes were identified by Takuno and Gaut (2012), and the status of body-methylation for 27 416 *Arabidopsis* genes was obtained from Lloyd et al. (2015).

**Step 3: Feature selection**. Two feature selection methods, the Student's *t*-test and the chi-square test, were, respectively, used to select numeric and binary features that have the capability of distinguishing positive samples and negative samples. The difference in the distribution of a given feature between positive and negative samples was deemed significant when the *P*-value was < 0.05. In this way, a total of 766 statistically significant features were identified (Table S3).

**Step 4: Random forest-based prediction model construction**. To implement the integrative random forest-based gene prioritization method RafSee, a prediction model was constructed using the random forest-based machine learning algorithm (Touw et al., 2013). This algorithm generated hundreds of decision trees built using a subset of samples and features randomly selected from a user-input feature matrix (positive and negative samples for training in rows, 766 selected features in columns). Using the trained prediction model, RafSee ranked candidate genes based on their probability to be a true flowering-time gene as estimated from votes from all the trees. The random forest algorithm was implemented using the R package “randomforest” (version 4.6-12; https://cran.r-project.org/web/packages/randomForest). The number of decision trees was set to ntree = 500 (all other parameters used default values).

### Network-based gene prioritization

The network-based gene prioritization was performed using the functional association network AraNet v2 web server (http://www.inetbio.org/aranet/), which was developed for identifying candidate genes of interests from *Arabidopsis* and 28 non-model plant species (Lee et al., 2015b). The functional associations between gene pairs (links) in AraNet v2 are inferred using a Bayesian statistics framework that integrates 19 distinct types of data: namely protein-protein interactions, co-expression, genomic context, domain co-occurrence, and phylogenetic profile similarity (Lee et al., 2015b). The integration of diverse biological data greatly improves network coverage and accuracy. Currently AraNet v2 consists of 895 000 co-functional links, covering 83.5% (22,894 out of 27,416) of all *Arabidopsis* protein-coding genes annotated in the TAIR10 database. The input of AraNet v2 is a set of seed genes of interest; the output is the rank of other genes in the network as determined by the co-functional prediction score of a Bayesian statistics framework.

### Order statistics-based meta-analysis

In RAP, an order statistics algorithm, known as robust rank aggregation, was applied to aggregate the prediction results from different gene prioritization methods (i.e., AraNet v2 and RafSeq). The robust rank aggregation is a powerful meta-analysis algorithm that uses a rank-order statistic for not only taking into account the positional information of input genes, but also for assigning a significance score (*P*-value) for each gene within a theoretical model (Aerts et al., 2006; Kolde et al., 2012). *M* is the number of candidate genes, and ***R***_*i*_ = (*r*_*i*, 1_, *r*_*i*, 2_, …, *r*_*i, n*_) is the vector of ranks for candidate gene *i* from different gene prioritization methods (here *n* = 2 for RafSee and AraNet v2). We first normalized gene ranks into percentiles ***U***_*i*_ = (*u*_*i*, 1_, *u*_*i*, 2_, …, *u*_*i, n*_) with the formula: *u*_*i, j*_ = *r*_*i, j*_/*M* (*j* = 1, 2, …, *n*). The *k*th smallest percentiles among *u*_*i*, 1_, *u*_*i*, 2_, …and *u*_*i, n*_is an order-statistic which follows a beta distribution *B*(*k, n* + 1 − *k*), under the assumption that the percentiles are uniformly distributed from 0 to 1. Based on the beta distribution, we then assigned a *P*-value to each percentile in ***U***_*i*_ indicating how much better it is ranked compared with a null model expecting random ordering. The significance score of the candidate gene *i* is defined as the minimum value of all *P*-values. The robust rank aggregation method was implemented using the R package “RobustRankAggreg” (version 1.1; https://cran.r-project.org/web/packages/RobustRankAggreg; Kolde et al., 2012).

### Implementation of RAP method

The RAP method has been implemented as an R package, which provides functions for generating sequence-based features (AAC, PAAC, APAAC, and PCP), and for extracting informative features with feature selection methods such as the student's *t*-test and chi-square test feature selection methods. Additionally, RAP provides functions to implement the integrative random forest-based gene prioritization method RafSee and to evaluate the prediction performance of gene prioritization methods with the cross-validation approach. To perform the gene prioritization in *Arabidopsis*, the user is only required to provide a set of genes of interest and the network-based gene prioritization results from the AraNet v2 system. With this data, RAP first ranks undocumented genes using the automatically built random forest-based gene prioritization method RafSee, and then it ranks the undocumented genes using the order statistics-based meta-analysis approach. The source code, sample data, and user manual of this R package are available at http://bioinfo.nwafu.edu.cn/software.

### Performance evaluation using a cross-validation algorithm

Cross-validation is a widely used evaluation method in machine learning for assessing the performance of prediction models. To evaluate the predictive performance of RafSee in distinguishing positives and negatives, we used the 10-fold cross validation algorithm and receiver operating characteristic (ROC) curve analysis. In a 10-fold cross-validation algorithm, positive and negative samples are randomly partitioned into 10 groups having an approximately equal number of genes; each group is successively used for testing the performance of RafSee trained with the other nine groups of positive and negative samples. For each round of cross-validation, the prediction accuracy of RafSee was assessed using the ROC-curve analysis, which measures how true positive rate (*y* axis) changes as function of the false positive rate (*x* axis) at all possible thresholds. The area under the ROC curve (i.e., AUC) was used to quantitatively score the prediction accuracy of RafSee. An AUC value can range from 0 to 1; a higher AUC value indicates better prediction accuracy for RafSee. After testing with each of the 10 groups, the mean value of the 10 AUCs represented the overall performance of RafSee.

The “leave-one-out” cross-validation technique was used to assess the prediction performance of different gene prioritization methods (i.e., AraNet v2, RafSee, and RAP) in ranking the flowering-time genes. In this technique, each flowering-time gene in the positive sample set was retained in turn as the testing sample while the remaining positive samples were used as the seed genes for three gene prioritization methods. The undocumented samples, negative samples, and the retained flowering-time gene were used as candidate genes for testing. A higher ranking of the retained flowering-time gene indicated a greater prediction accuracy of the gene prioritization method(s).

## Results

### Sequence, evolutionary, and epigenetic characteristics of flowering-time genes

**Figure 2:**
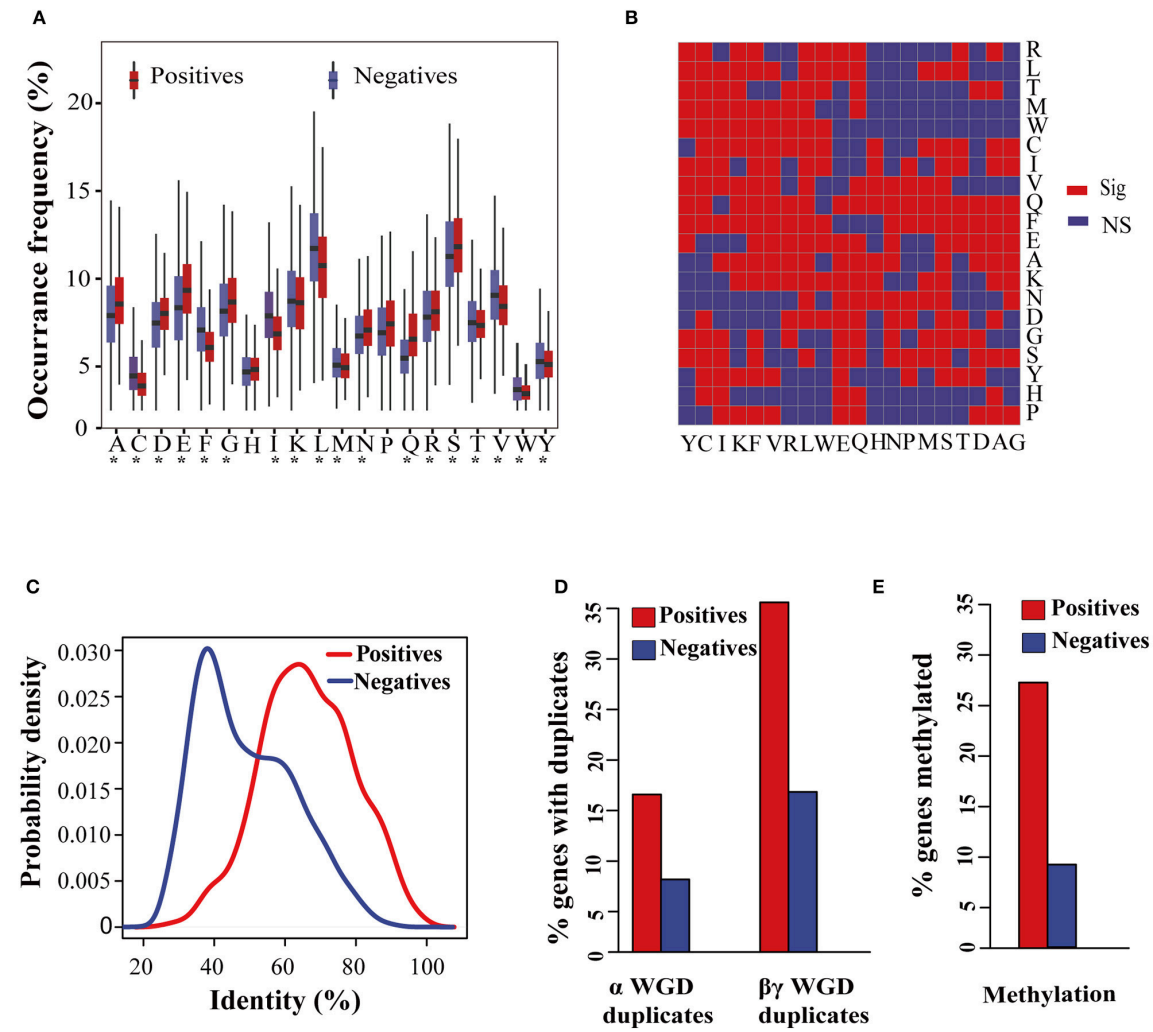
**Distribution of sequence, evolutionary, and epigenetic features in the positive and negative sample sets. (A)** Boxplot distributions for the occurrence frequency of 20 amino acids in the positive and negative sample sets. Asterisks (^*^) indicate that the differences between positive and negative samples are statistically significant at the level of 0.05. **(B)** Differences in the occurrence frequency of 400 amino acid pairs between positive and negative samples. “Sig” represents a significant difference and “NS” represents a non-significant difference at the level of 0.05. **(C)** Density distributions of the median percentage of identity of positive and negative samples to the top BLASTP matches in 34 plant species. **(D)** Percentage of positive and negative samples that have a paralog derived from α and βγ whole genome duplicates. **(E)** Percentage of genes with methylation in the positive and negative sample sets.

We first generated 1012 features for each protein sequence of 27,416 *Arabidopsis* genes (449 positive samples, 8503 negative samples, and 18,464 undocumented samples), and then identified 766 features that differed between the positive and negative samples at a significance level of 0.05 (Table S3). Among these 766 features there were 255 ACC-related features, including the occurrence frequency of 18 amino acids and 237 amino acid pairs (Figures 2A,B). Besides the occurrence frequency of amino acids, we also noticed that the order of amino acids in flowering-time genes was not completely random. For example, seven of 20 amino acid pairs starting with histidine (H) were significantly different in their occurrence frequency between positive and negative samples, while five of 20 amino acid pairs ending with histidine (H) showed significant differences (Figure 2B).

We also detected significant differences for hydrophilicity and hydrophobicity patterns of protein sequences corresponding to six APAAC-related features; these included the third-order factor in term of hydrophilicity of amino acids, the first-order correlation factor, the second-order correlation factor, up to the fifth-order factor in term of hydrophobicity of amino acids.

The PCPs are a group of essential features for characterizing physicochemical properties of protein sequences. For this reason PCPs have been widely used in the prediction of protein structure, functional sites, and biological functions because of their interpretability (Mallick et al., 2007; Li et al., 2013; Chaudhary et al., 2015). Here, 462 out of 533 PCP-related features were significantly different between positive and negative samples (Table S3). Among the top 10 PCP-related features ranked by level of statistical significance, five were related to the hydrophobicity of amino acids as calculated with different measures (top 3, 4, 6–8; Table 1). Another three of the top 10 PCP-related features were energy-related features, including that for the free energy of transfer of amino acids from organic solvent to water (top 1; Nozaki and Tanford, 1971), the contribution of amino acids to the stability of proteins (top 2; Zhou and Zhou, 2004), and the energy required to transfer amino acid side chains from water to less polar environments (top 9; Guy, 1985). There were also two PCP-related features involved in the retention coefficients of different amino acids in both NaH_2_PO_4_ and NaClO_4_ (top 5, 10; Meek and Rossetti, 1981).

**Table 1:** **List of the top 10 PCP-related features**.

**Rank**	**AA index ID**	**Description**	***P*-value**	**References**
1	NOZY710101	Transfer energy, organic solvent/water	1.16E-86	Nozaki and Tanford, 1971
2	ZHOH040101	The stability scale from the knowledge-based atom-atom potential	7.16E-86	Zhou and Zhou, 2004
3	SWER830101	Optimal matching hydrophobicity	5.04E-83	Sweet and Eisenberg, 1983
4	CORJ870102	SWEIG index	6.67E-83	Cornette et al., 1987
5	MEEJ810102	Retention coefficient in NaH_2_PO_4_	9.77E-82	Meek and Rossetti, 1981
6	CIDH920104	Normalized hydrophobicity scales for alpha/beta-proteins	5.62E-81	Cid et al., 1992
7	CIDH920103	Normalized hydrophobicity scales for alpha + beta-proteins	5.74E-80	Cid et al., 1992
8	CIDH920105	Normalized average hydrophobicity scales	8.34E-80	Cid et al., 1992
9	GUYH850102	Apparent partition energies calculated from Wertz-Scheraga index	1.82E-79	Guy, 1985
10	MEEJ810101	Retention coefficient in NaClO_4_	2.20E-79	Meek and Rossetti, 1981

Altered flowering time has been suggested as an evolutionary strategy adopted by plants to quickly adapt to different environments (Kazan and Lyons, 2016). Therefore, we suspect that differences may exist in the evolutionary patterns between positive and negative samples. The SC measures the identity of an *Arabidopsis* protein against protein sequences from 34 other plant species (see Section Materials and Methods). As shown in Figure 2C, flowering-time genes have, on average, a 65% shared identity with those of the other 34 species, while the negative samples have just about a 40% identity.

During the evolutionary process, the *Arabidopsis* genome has experienced at least two ancient whole-genome duplication (WGD) events (α WGD and βγ WGD; Yun et al., 2012). With the sequenced genome, 6830 and 2896 *Arabidopsis* genes with paralogs derived from α and βγ WGD events were identified, respectively (Yun et al., 2012). We found that over 15 and 30% flowering-time genes have a paralog derived from α and βγ WGD events, respectively (Figure 2D). Nevertheless, as for the negative samples, this amounted to <10 and 20% of those that occurred in these two WGD events.

Following the hypothesis that body-methylated genes would be more functionally important than non-methylated genes (Coleman-Derr and Zilberman, 2012), we examined the percentage of body-methylated genes in the positive and negative sample sets. We found 27.17% (122/449) flowering-time genes that were body-methylated, whereas only 8.49% (722/8503) genes were body-methylated in the negative sample set (Figure 2E). This result supports the view that changes in the epigenome are important in regulating the flowering time of plants (Yaish et al., 2011).

### Performance evaluation of RafSee in distinguishing positives and negatives

**Figure 3:**
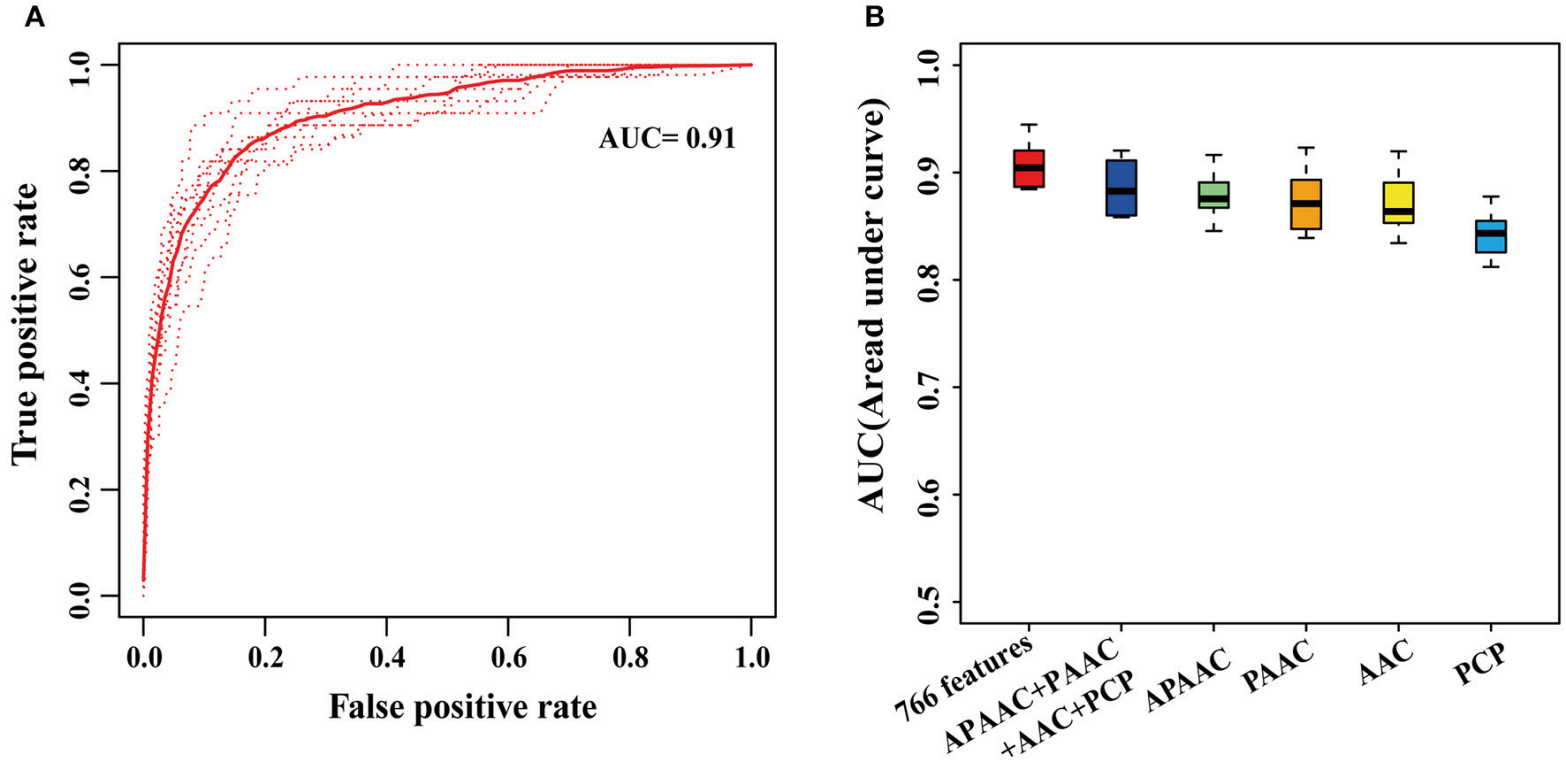
**Performance of RafSee in distinguishing positives and negatives using 10-fold cross validation. (A)** The ROC curves of 10-fold cross validation for RafSee trained with 766 statistically significant features. The dashed curves denote the ROC curves from the testing dataset in each round of 10-fold cross-validation. The solid curves represent the average curve of the 10 ROC curves. **(B)** Boxplot distribution of 10 AUC values of the 10-fold cross validation for RafSee trained with different sets of features. The APAAC, PAAC, AAC, and PCP, respectively indicated 26 APAAC-, 20 PAAC-, 255 AAC-, and 461 PCP-related statistically significant features extracted from protein sequences.

Using 766 statistically significant features with *P* < 0.05, we presented a novel integrative random forest-based gene prioritization method named RafSee, the prediction performance of which was evaluated with 10-fold cross validation and ROC analysis. In Figure 3A are shown the ROC curves of RafSee trained with 766 features, while Figure 3B presents the distribution of 10 AUC values generated from the 10-fold cross validation for RafSee that was trained with different sets of statistically significant features. We found that RafSee trained with 461 PCP-related statistically significant features had a mean AUC value of 0.84 (Figure 3B). In contrast, RafSee trained with 26 APAAC-, 20 PAAC-, or 255 AAC-related statistically significant features could more accurately distinguish positive and negative samples, as suggested by a higher mean AUC value of ~0.87 (Figure 3B). The mean AUC value reached 0.89 when all these statistically significant features extracted from protein sequences were considered (Figure 3A). The mean AUC value can be further improved from 0.89 to 0.91 by integrating these protein sequence-based features with four additional features (one SC-related feature, two WGD-related features plus one methylation-related feature; Figure 3A).

Taken together, these results suggest that RafSee significantly outperformed the random selection (i.e., AUC = 0.5) in the identification of flowering-time genes.

### Performance comparison of AraNet v2, RafSee, and RAP in the prioritization of flowering-time genes

**Figure 4:**
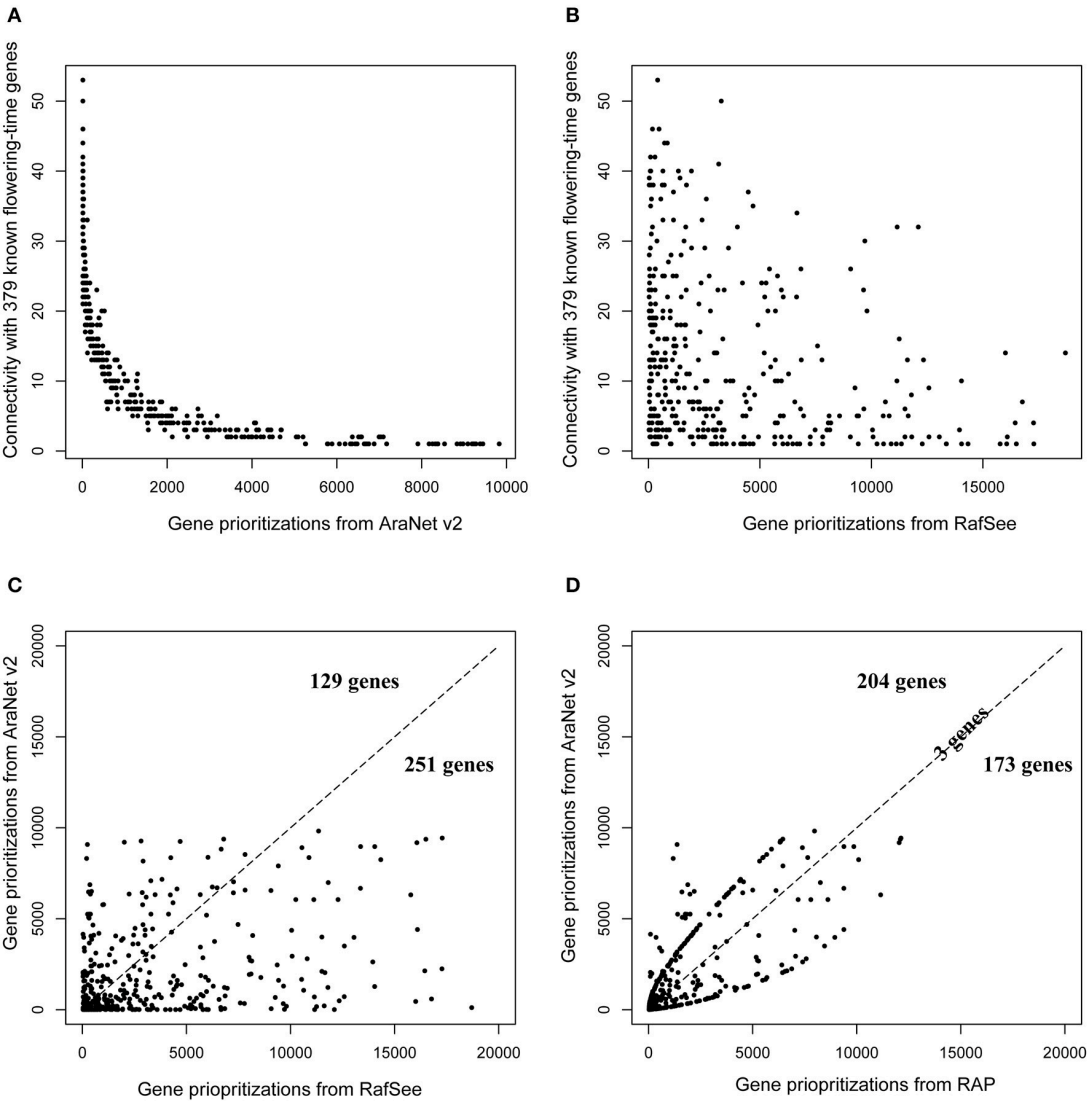
**Performance of three different gene prioritization methods for identifying flowering-time genes. (A)** Relationships between gene rank and their connectivity with known flowering-time genes for AraNet v2. **(B)** Relationships between gene rank and their connectivity with known flowering-time genes for RafSee. **(C)** Pairwise comparison between gene ranks predicted by AraNet v2 and RafSee. Each symbol denotes a flowering-time gene, and its coordinates represent the ranks assigned by the corresponding two gene prioritization methods. The dashed diagonal line denotes a 1:1 correspondence. **(D)** Pairwise comparison between gene ranks predicted by AraNet v2 and RAP.

The “leave-one-out” cross-validation experiment was first employed to evaluate the performance of the network-based gene prioritization system (AraNet v2). We found that 380 of 449 (84.63%) flowering-time genes can be prioritized by the AraNet v2 system (Table S4). For a fair comparison, these 380 flowering-time genes were also used to perform the “leave-one-out” cross-validation experiment for the other two gene prioritization methods (RafSee and RAP). We observed that genes tend to be ranked higher by AraNet v2 when they are connected to more known flowering-time genes in the network (Figure 4A). However, this trend was not observed for RafSee (Figure 4B). This is expected, as the AraNet v2 system uses the edge-based network properties for gene prioritization, while RafSee not. The agreement between the ranks of these 380 flowering-time genes prioritized by AraNet v2 and RafSee was very low, with the Spearman correlation coefficient of 0.31(Figure 4C; Table S4), and 33.94% (129/380) of the genes prioritized by RafSee had a higher rank than given by AraNet v2 (Figure 4C).

These results indicate that the integrative random forest-based gene prioritization method (RafSee) could be used as a complement to the network-based gene prioritization method (AraNet v2). As such, it provided an opportunity for us to present a novel integrative approach (RAP) for improving gene prioritization by aggregating gene ranks produced by these two different gene prioritization methods. We found that RAP improved the rank of 53.68% (204 of 380) flowering-time genes (Figure 4D). We further evaluated the performance of three gene prioritization methods using different ranking statistics: namely the minimum, first quartile, median, third quartile, and maximum rank (Table 2). For all these statistics, AraNet v2 had higher ranks than RafSee for identifying flowering-time genes. However, by utilizing the complement between these two gene prioritization methods, RAP obtained the best results for all these ranking statistics (except the maximum rank). For example, RAP obtained the first quartile rank of 90.5, whereas AraNet v2 and RafSee had corresponding values of 149.5 and 415.5, respectively. We note that feature selection is an important factor to affect the performance of RafSee and RAP. For example, using the fairly strict feature selection criteria of *P* < 0.01, RafSee and RAP showed a slightly decreased performance, corresponding to the first quartile rank of 419.75 and 116.5, respectively (Table S5). Even so, RAP still obtained the best results for the minimum, first quartile, and third quartile rank.

**Table 2:** **Performance statistics for ranking flowering-time genes using different gene prioritization methods**.

**Methods**	**Minimum**	**First quartile**	**Median**	**Third quartile**	**Maximum**
RafSee	7	415.5	1908.5	5419.5	18678
AraNet v2	**1**	149.5	830	3019.25	**9817**
RAP	**1**	**90.5**	**743**	**2508.25**	12099

*Bold denotes the best method for the corresponding ranking criteria*.

These results demonstrate that the integrative analysis further improved the performance of single gene prioritization methods (i.e., AraNet v2 and RafSee).

### Validation of the RAP-based gene prioritization with network analysis and evidence from the literature

**Figure 5:**
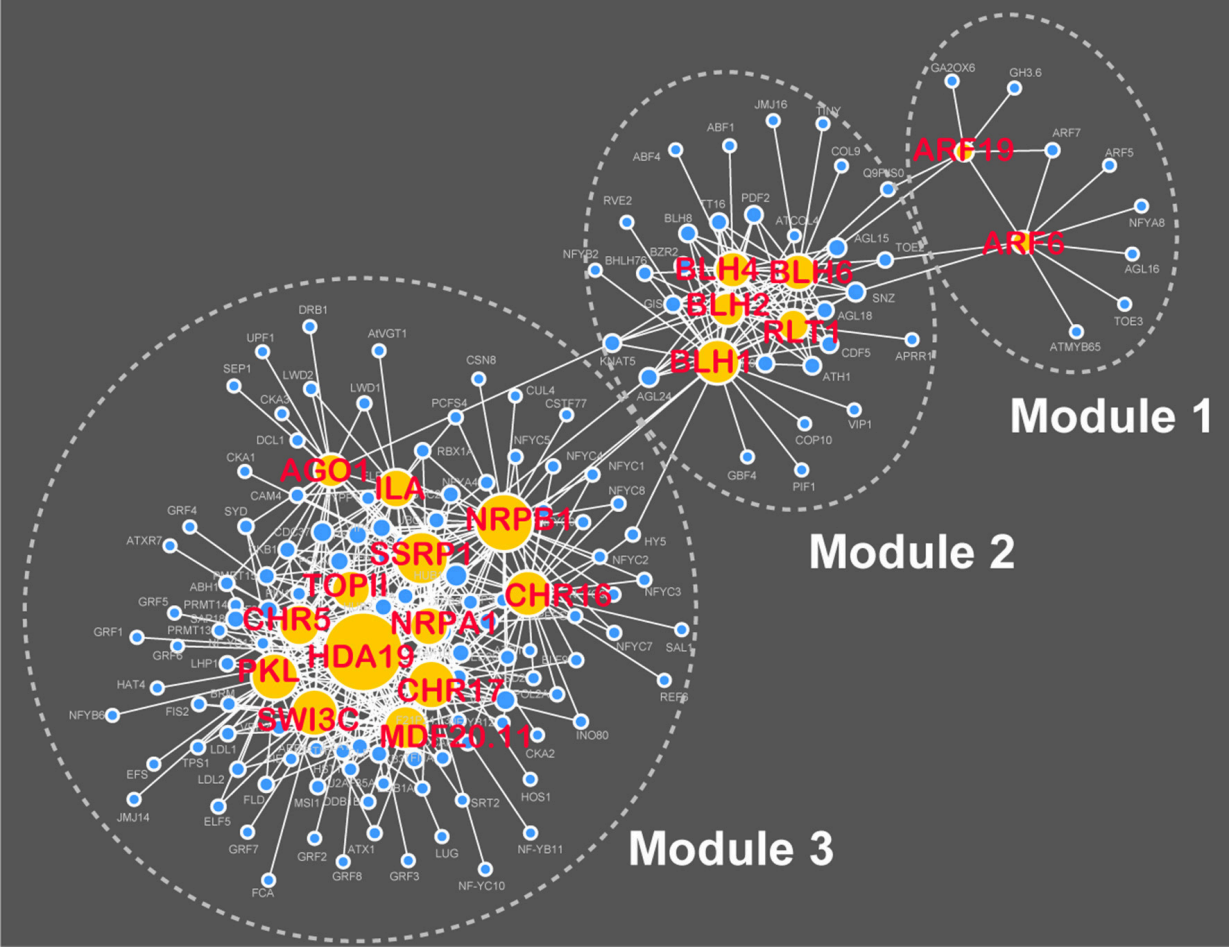
**A hierarchical network of functional associations between the top 20 ranked genes and 449 known flowering-time genes**.

With the input of 449 flowering-time genes, RAP was applied to rank the remaining 26 968 genes annotated in the TAIR10 database (Table S5). Further, network analysis revealed that the top 20 ranked genes connect with 150 known flowering-time genes in the AraNet v2 system, resulting in the generation of a hierarchical network that contains three modules and 418 functional associations (Figure 5; Table S6). This result indicates that the top 20 candidates identified by RAP might be functionally associated with flowering time in *Arabidopsis*. To validate the new candidate genes identified by the RAP method, we performed the linkage disequilibrium analysis of a flowering-time-related genome-wide association study dataset (Atwell et al., 2010) using the TASSEL software (http://www.maizegenetics.net/tassel). The linkage disequilibrium plots also showed potentially functional associations of the top 20 ranked genes with flowering time in *Arabidopsis* (Figure S1). In addition, through a literature review, we found that nine of the top 20 candidates (AT2G25170, AT2G23760, AT1G21700, AT1G19220, AT4G36870, AT4G38130, AT1G28420, AT5G18620, and AT1G48410) have been recently demonstrated to have roles in the control of flowering time with phenotype experiments (Table S7). From these results, we conclude that RAP should be reliable and effective to prioritize large numbers of candidate genes in *Arabidopsis*.

### Evaluating the accuracy of RafSee, AraNet v2, and RAP with stress-related genes

We further performed the “leave-one-out” cross-validation experiment to evaluate the performance of AraNet v2, RafSee, and RAP in prioritizing *Arabidopsis* genes related to different abiotic stresses (salt, water, cold, and temperature). Stress-related genes were obtained from Gene Ontology (GO) database (http://geneontology.org) by exacting terms (i.e., response to salt/water/cold/temperature) annotated with an experimental evidence code IDA (inferred from direct assay), IEP (inferred from expression pattern), IGI (inferred from genetic interaction), IPI (inferred from physical interaction), and/or IMP (inferred from mutant phenotype). The six positive sample sets contained 388, 373, 289, and 238 genes that were mostly experimentally validated to be related to salt, temperature, cold, and water stresses, respectively.

Table 3 lists the evaluation results of AraNet v2, RafSee, and RAP in terms of five ranking criteria (the minimum, first quartile, median, third quartile, and maximum rank). In the prioritization of salt- and temperature-related genes, RAP outperformed RafSee and AraNet v2 for all these ranking statistics (except the maximum rank). While prioritizing cold-related genes, RafSee had the best result only for the first quartile rank, AraNet v2 had the best results for the third quartile and maximum rank, RAP had the best results for the minimum and first quartile rank. Tests on the water stress-related gene set showed that RAP outperformed RafSee and AraNet v2 in terms of both first quartile and median rank.

**Table 3:** **Performance statistics for ranking stress-responsive genes using different gene prioritization methods**.

**Stress-responsive genes**	**Methods**	**Minimum**	**First quartile**	**Median**	**Third quartile**	**Maximum**
Response to salt (388 genes)	RafSee	6	1035.5	8928.75	8928.75	19202
	AraNet v2	11	1088	6131.5	6131.5	**11539**
	RAP	**4**	**1011.5**	**6103**	**6103**	13130
Response to temperature (373 genes)	RafSee	**1**	1270	3520	8623	19550
	AraNet v2	**1**	1025	2668	5810	**11319**
	RAP	**1**	**933**	**2532**	**5667**	13029
Response to cold (289 genes)	RafSee	10	**870**	3423	9165	21571
	AraNet v2	9	1282	2832	**5217**	**10416**
	RAP	**4**	916	**2414**	5542	12355
Response to water (238 genes)	RafSee	**1**	1026.25	3712.5	8260.25	22045
	AraNet v2	18	754	2155	**4569**	**9908**
	RAP	8	**626.25**	**1783**	5070.25	12007

*Bold denotes the best method for the corresponding ranking criteria*.

## Discussion

The number of available genome sequences and gene networks is steadily increasing in the field of plant biology. Network-based gene prioritization approaches has been widely applied to identify new genes involved in biological processes of interests (Li et al., 2015), such as abiotic stress responses (Ma et al., 2014; Sircar and Parekh, 2015), secondary wall formation (Ruprecht et al., 2011), glucosinolate secondary metabolism (Chan et al., 2011), and plant growth (Sabaghian et al., 2015). In this study, we presented an integrative random forest method called RafSee and a meta-analysis based approach called RAP to prioritize genes from a large set of candidates. We validated the predictive power through the “leave-one-out” cross-validation approach in five different case studies including flowering time and four stress-related studies (salt, cold, water, and temperature). All these studies showed that RafSee can be used as a complement to a current state-of-art network-based gene prioritization system (AraNet v2). Moreover, RAP can be used to improve the performance of the network-based gene prioritization system. We anticipate that RAP will accelerate the discovery of genes involved in many biological processes and plant traits of interest.

RAP has several inherent advantages compared with the network-based gene prioritization methods. First, instead of using edge-based network properties, RafSee builds gene prioritization models using features exacted from protein sequences, evolutionary conservation, and epigenetic methylation marks. This allowed RafSee to rank 69 flowering-time genes that failed to be ranked by the AraNet v2 system (Table S4). Second, the order statistics-based meta-analysis approach can be used to effectively aggregate the rank of RafSee and the network-based gene prioritization system AraNet v2. While prioritizing flowering-time genes, RAP improved the performance of AraNet v2 from 149.5 to 90.5, resulting in an 39.46% improvement in term of the first quartile rank. Last, the RAP method has been implemented as an R package, providing a flexible framework for aggregating gene prioritizations from different types of biological networks. Besides the functional association networks (e.g., AraNet v2 and STRING), co-expression networks capture the functional relationships between genes solely from gene expression datasets, which can also be integrated in RAP for gene functional analysis in several crop species, including maize, rice, soybean and wheat (Mutwil et al., 2011; Aoki et al., 2016; Ruprecht et al., 2016; Serin et al., 2016).

Nonetheless, we are also aware of several limitations to our proposed method. First, feature selection is applied to select a set of informative features, which may affect the performance of RafSee and RAP (Table 2, Table S5). Second, the power of RafSee and RAP is affected by the size of seed genes. We performed 140 simulation experiments to examine the performance of three gene prioritization methods trained with a varied size of seed genes (350, 300, 250, 200, 150, 100, and 50 randomly selected flowering genes; 10 replications per gene size). We found that RAP improved the performance of AraNet v2 in term of the third quartile rank in the majority of simulation experiments (73%; 73/100), when the size of seed genes was equal to or higher than 150. However, for the same statistic criteria, RAP improved the performance of AraNet v2 in only 35% (14/40) simulation experiments, when the size of seed genes is <150 (Supplementary Data 2).

In the future, we plan to investigate the effectiveness of RAP in gene prioritization using different biological networks and machine learning algorithms. Finally, we want to expand the application of RAP from model species to crop species.

## Author contributions

Designed the experiments: JZ, CM. Performed the experiments: JZ, HY, and LW. Analyzed the data: JZ, YT, CM, and HS. Wrote the paper: CM, JZ, and YT. All authors read and approved the final manuscript.

## Funding

This work was supported by the National Natural Science Foundation of China (31570371), the Agricultural Science and Technology Innovation and Research Project of Shaanxi Province, China (2015NY011), and the Fund of Northwest A&F University.

### Conflict of interest statement

The authors declare that the research was conducted in the absence of any commercial or financial relationships that could be construed as a potential conflict of interest.

## Supplementary material

Table S1 **The 449 known flowering time-related genes**.

Table S2 **The 533 physicochemical properties of 20 amino acids**.

Table S3 **Significance estimation of 1012 features generated with different encoding schemes**.

Table S4 **The “leave-one-out” cross-validation results of collected flowering-time genes using different gene prioritization methods**.

Table S5 **Performance statistics for ranking 380 flowering-time genes using different gene prioritization methods with fairly strict feature selection criteria of ***P*** < 0.01**.

Table S6 **Functional associations between top 20 ranked genes and known flowering-time genes**.

Table S7 **Interpretation of the top 20 ranked genes related to flowering time in ***Arabidopsis***. The top 20 genes identified by RAP, and their potential roles in flowering time from a survey of the literature**.

Supplementary Data 1 **Definition of pseudo amino acid composition (PAAC) and amphiphilic pseudo amino acid composition (APAAC)**.

Supplementary Data 2 **Performance of three gene prioritization methods affected by the size of seed genes**.

Figure S1 **Linkage disequilibrium plots for top 20 candidate genes**.
