# Supplementary material for: A Meta-Analysis Based Method for Prioritizing Candidate Genes Involved in a Pre-specific Function
Source: Front Plant Sci. 2016 Dec 15;7:1914. doi: 10.3389/fpls.2016.01914 (PMC5156684; doi:10.3389/fpls.2016.01914)
Supplement: Supplementary Data 1 — Definition of pseudo amino acid composition (PAAC) and amphiphilic pseudo amino acid composition (APAAC). [file DataSheet1.DOCX]

Supplementary Material

A meta-analysis based method for prioritizing candidate genes involved in a pre-specific function

**Jingjing Zhai*****^†^*, Yunjia Tang*^†^*, Hao Yuan, Longteng Wang, Haoli Shang, Chuang Ma^*^**

*** Correspondence:** Chuang Ma: chuangma2006@gmail.com

# Supplementary Data

**Pseudo amino acid composition (PAAC)**

For a given protein with sequence length ***L***, PAAC can be depicted as:

- $\boldsymbol{P}= \left[ p_{1}, p_{2},\ldots, p_{20},\ldots, p_{20+1}, p_{20+2},\ldots, p_{20+\lambda} \right] (\lambda<L)$,

where the component in ***P*** is calculated by:

- $P_{u}=\left\{ \begin{aligned} \frac{f_{u}}{\sum_{i=1}^{20} f_{i}+\omega\sum_{k=1}^{\lambda} \tau_{k}}, & 1 \leq u \leq20 \\ \frac{\omega\tau_{u-20}}{\sum_{i=1}^{20} f_{i}+\omega\sum_{k=1}^{\lambda} \tau_{k}}, & 20+1 \leq u \leq20+\lambda\end{aligned} \right.,$

here $\omega$ is the weight factor and $\tau_{k}$ is the correlation factor that reflects the sequence order correlation between all the *k*-th most contiguous residues as formulated as by:

- $\tau_{k}= \frac{1}{3(L-k)}\sum_{i=1}^{L-k} \left\{ \left[ H_{1}(R_{i+k})-H_{1}(R_{i}) \right]^{2}+ \left[ H_{2}(R_{i+k})-H_{2}(R_{i}) \right]^{2}+\left[ M(R_{i+k})-M(R_{i}) \right]^{2} \right\}$,

where $H_{1}(R_{i})$, $H_{2}(R_{i})$ and $M(R_{i})$ represent the normalized hydrophobicity value, hydrophilicity value and the side chain mass for amino acid residue $R_{i}$, respectively; while $H_{1}\left( R_{i+k} \right), H_{2}\left( R_{i+k} \right) andM_{1}(R_{i+k})$ are those for amino acid residue $R_{i+k}$.

**Amphiphilic pseudo amino acid composition (APAAC)**

In the amphiphilic mode, the given protein ***P*** can be represented as:

- $P= \left[ p_{1}, p_{2},\ldots, p_{20},\ldots, p_{20+1}, p_{20+2},\ldots, p_{20+2\lambda} \right](\lambda<L)$,

where the $20+2\lambda$ components are calculated by:

- $P_{v}=\left\{ \begin{aligned} \frac{f_{v}}{\sum_{i=1}^{20} f_{i}+\omega\sum_{j=1}^{2\lambda} \tau_{j}}, & 1 \leq v \leq20 \\ \frac{\omega\tau_{v-20}}{\sum_{i=1}^{20} f_{i}+\omega\sum_{j=1}^{2\lambda} \tau_{j}}, & 20+1 \leq v \leq20+2\lambda\end{aligned} \right.$,

here $\tau_{j}$ can be represented as follows:

- $\tau_{m}= \frac{1}{L-l}\sum_{i=1}^{L-l} h^{1}\left( R_{i} \right)h^{1}(R_{i+1})$,
- $\tau_{n}= \frac{1}{L-l}\sum_{i=1}^{L-l} h^{2}\left( R_{i} \right)h^{2}(R_{i+1})$,

where $m=1, 3, 5, \ldots, (2\lambda-1)$; n$=2, 4, 6, \ldots, 2\lambda$ and *l*$=1, 2, 3, \ldots, \lambda$($\lambda<L$), $h^{1}\left( R_{i} \right)$ and $h^{2}\left( R_{i} \right)$ represent the normalized hydrophobicity and hydrophilicity properties of amino acid residue $R_{i}$, respectively; while $h^{1}(R_{i+1})$ and $h^{2}(R_{i+1})$ are those amino acid residue $R_{i+1}$.
