# Supplementary figures and images for: A Meta-Analysis Based Method for Prioritizing Candidate Genes Involved in a Pre-specific Function
Source: Front Plant Sci. 2016 Dec 15;7:1914. doi: 10.3389/fpls.2016.01914 (PMC5156684; doi:10.3389/fpls.2016.01914)

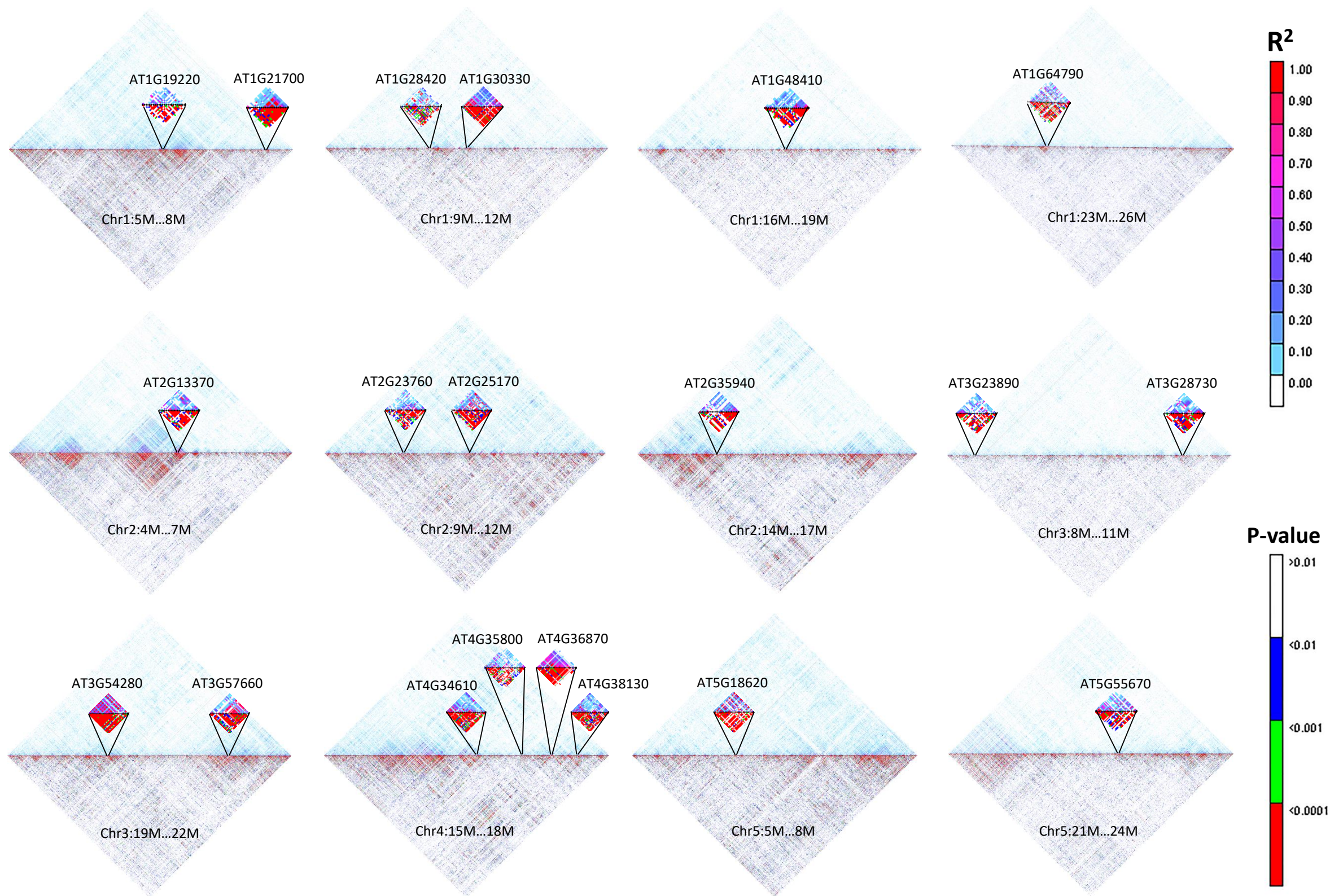

Supplement: Figure S1 — Linkage disequilibrium plots for top 20 candidate genes. [file Image1.PDF]
